# Monthly Summary

**Published:** 1879-06

**Authors:** 


### MONTHLY SUMMARY.

---

*Rapid Anæsthesia.*—Give the patient the ether-inhaler, let him hold it to his face with one hand and elevate the other. In a few minutes the arm will drop, and there will be from thirty to fifty seconds of unconsciousness, during which minor operations of surgery, reduction of dislocations, etc., can be performed. The right moment must be seized, for, if the patient returns to consciousness, full etherization will then have to be employed. —*Phil. Med. Times.*

*Mal-Practice Case.*—A case of prosecution for mal-practice, in which a Miss Brooks, of Columbus, Ohio, was plaintiff, and Dr. Todd, D. D. S., of the same city, was defendant, has after a trial lasting thirteen days, been decided adverse to the defendant, judgment being obtained against him for nine hundred and twenty-five dollars.

The operation involved in the case was an attempt to extract an inferior third molar, in which the tooth seems by some means to have escaped the grasp of the instrument used, and passed into the soft tissues on the inner side of the jaw near to its angle, and remaining there for several weeks; it was alleged to have been the cause of extensive disease in the parts, and great suffering and prostration. This, it is presumed, was proven in court.

The tooth, after several weeks lodgment, was removed by another operator.

Of the merits of this case I know but little, having never seen it, and have had only indefinite descriptions of it.

It is quite possible that the manipulation for the removal may have been faulty, or slight inattention on the part of the operator may have been an element in the disaster, or there may have been no fault in either of these respects, and yet to the struggling and resistance of the patient, the accident may have been wholly due; to such an extent does this sometimes occur that the wonder is, that serious accidents do not more frequently take place.

In this instance it is certain that, from some cause or other, a firm and proper grasp was not made upon the tooth with a proper instrument, or it could not have escaped as it did.

This case contains another lesson to the members of our profession, viz: that they should better understand the legal relation that exists between them and their patients; that they should in all doubtful cases put themselves in a safe position before proceeding to operate.

Then again, it should be a stimulus to those seeking to enter the profession, to make themselves most thoroughly familiar with the best modes of practice, and be able to put them into execution.

There are very few, if any, in the practice of dentistry or surgery, who might not, through the machination of mischievous attorneys and the ill advice of friends, be entangled in the meshes of mal-practice suits, not perhaps in many instances with the results of the above case, but any such entanglement is disastrous, whatever the issue may be.

In every such case the members of the profession should come to the rescue, unless it be manifestly a case of mal-practice, and in some such cases money can not make sufficient restitution.

If mal-practice suits are encouraged upon every frivolous pretense, then no one knows when he is safe.

Let the profession demand of its own members the largest measure of attainments and ability, and then stand solid as one man against unjust prosecutions for alleged mal-practice.—*Dental Register.*

---

*Chewing Gum.*—Among the quiet little manufactures of the country is that of chewing gum. One factory exists in this city, and others are in New England, New York State, Ohio, Illinois, and Tennessee. The gum is sold by druggists, grocers, and confectioners in cities. Gum from spruce trees was exclusively used until recently, when it found a rival in so-called gum mastic (not real gum mastic,) a white and attractive article made from paraffine, which is sweetened. The consumption of this chewing gum in the United States is about thirty tons yearly; that of spruce gum somewhat less, and that of a gum made in Tennessee, from balsam tolu, and sold in the Southern States, about twenty tons. Lately a material has been used styled "rubber gum." It is from the sap of the sapote tree of South and Central America. The sap, like that of the India rubber tree, has a milky look. The gum was first imported into the United States with a view of melting it with India rubber, in order to produce a cheaper article than the latter. It was found to be impliable, and therefore useless for that purpose. It had long been chewed by South and Central American Indians, and found useful in allaying thirst. Experiments were therefore made here in purifying it for chewing, and with final success. It is tasteless, and has the merit of lasting longer than other gums, which more quickly dissolve and crumble in the mouth. So great is its ductility that a piece half an inch square, after being heated in the mouth, can be stretched into a thread a hundred feet long. Its consumption is about fifty tons a year. Chewing gum does not, like tobacco, require that the saliva shall be expectorated; it does not, like smoking, excite the nerves, nor, like a superabundance of food or drink, hurtfully overload the stomach.—*Scientific News.*

---

*A Remarkable Old Man.*—There lives in Machias, Me., a man named John Hughes, who is, now in his 98th year, and who is hale and hearty, working every day, Sundays excepted. Sixteen years ago his eyesight became renewed, and is now as good as when he was young. He has lately had four new natural teeth come in place of those lost many years ago. He attends every town meeting, having voted at every presidential election from Washington to Hayes. He is a pensioner of the war of 1812. He reared a family of 16 children—8 boys and 8 girls—all living to be men and women. Two of his sons reside in Aroostook County, one in Houlton and one in Mapleton. Six of his sons were in the Union army during the rebellion, all of whom were officers.—*Boston Journal.*

*Chloroform Accidents.*—Many chloroform accidents are doubtless due to impurities in the drug. A French chemist, M. Perrin, states that commercial chloroform has become much less reliable and more dangerous of late years. Sleep is often difficult to get with it, and he mentions some cases in which the attempt had to be given up, after trying successfully the drug procured in several shops. It often produces disorder of the stomach, (moreover, vomiting, etc.,) and twice in his recent experience it caused a state of apparent death, which was followed by extreme exhaustion.

If chloroform, when tested by the addition of sulphuric acid, turns a fine red mahogany tint, it contains impurities and should not be used.—*Med. and Surg. Reporter.*

---

*How to Care for the Eyes.*—According to Dr. Javal, of Paris, the increase of near-sightedness in France is due to the over-exertion and fatigue of the eyes. It is well known that after having looked fixedly for some time at a piece of checkered stuff, the sight becomes troubled, the eye being fatigued, through the repetition of the same colors. Now, the same thing will occur in reading a book which rests on the table; if, however, it be moved up and down, the cause of fatigue will be removed. Another origin of fatigue to the eye is the black lines on white ground, such as they exist in almost all the books. The eye is not achromatic; and if the blue color of the solar spectrum be suppressed, the spectrum of diffusion on the retina is to a certain extent avoided, which relieves the eye greatly. Therefore, the printing paper ought to be of a yellowish (wash leather) color. Another cause of fatigue is the length of the printed lines on wide pages of one column.—*Med. and Surg Reporter.*

---

*Action of Substances on the Teeth.*—As the result of numerous trials made by the exposure of recently extracted teeth to the action of various substances, M. Maurel comes to the conclusion (in the *Jour. de Therapeutique*) that if various medicinal substances are dangerous in their action on the teeth, others in still larger numbers prove, in their habitual employment, quite inoffensive. Thus, if we are required to take great precautions respecting citric acid, tannin, chlorides of zinc and antimony, perchloride of iron, iodine, sulphate of copper and alum, we may continue to employ with complete safety arsenious and carbolic acids, vinegar, corrosive sublimate, chlorate of potash, alcohol, tincture of benzoin, essence of mint, tincture of quinine, and eau de Cologne. Tobacco, whether used in chewing or smoking, does not injure the teeth beyond causing their discoloration.—*Med. and Surg. Reporter.*

